# Early dissemination of bevacizumab for advanced colorectal cancer: a prospective cohort study

**DOI:** 10.1186/1471-2407-11-354

**Published:** 2011-08-16

**Authors:** S Yousuf Zafar, Jennifer L Malin, Steven C Grambow, David H Abbott, Deborah Schrag, Jane T Kolimaga, Leah L Zullig, Jane C Weeks, Mona N Fouad, John Z Ayanian, Robert Wallace, Katherine L Kahn, Patricia A Ganz, Paul Catalano, Dee W West, Dawn Provenzale

**Affiliations:** 1 Division of Medical Oncology, Department of Medicine, Duke University Medical Center, Durham, NC, USA; 2 Center for Health Services Research in Primary Care, DDurham VA Medical Center, Durham, NC, USA; 3 Greater Los Angeles VA Healthcare System, Los Angeles, CA, USA; 4 Division of Hematology and Medical Oncology, Department of Medicine, David Geffen School of Medicine, University of California, Los Angeles, Los Angeles, CA, USA; 5 Department of Biostatistics and Bioinformatics, Duke University Medical Center, Durham, NC, USA; 6 Dana-Farber Cancer Institute, Boston, MA, USA; 7 Division of Gastroenterology, Department of Medicine, Duke University Medical Center, Durham, NC, USA; 8 University of North Carolina at Chapel Hill, Department of Health Policy and Management, Chapel Hill, NC, USA; 9 Department of Health Policy and Management, Dana Farber Cancer Institute, Boston, MA, USA; 10 Division of Preventative Medicine, University of Alabama at Birmingham, Birmingham, AL, USA; 11 Department of Health Care Policy, Harvard Medical School, and Division of General Medicine, Brigham and Women's Hospital Boston, MA, USA; 12 Department of Epidemiology, College of Public Health, University of Iowa, Iowa City, IA, USA; 13 RAND Corporation, Santa Monica, CA; and Divisions of General Internal Medicine & Health Services Research, Department of Medicine, University of California Los Angeles, Los Angeles, CA, USA; 14 School of Public Health, University of California, Los Angeles, CA, USA; 15 Department of Biostatistics and Computational Biology, Dana Farber Cancer Institute, Boston, MA, USA; 16 Northern California Cancer Center, Fremont, CA; and Stanford School of Medicine, Stanford, CA, USA

## Abstract

**Background:**

We describe early dissemination patterns for first-line bevacizumab given for metastatic colorectal cancer treatment.

**Methods:**

We analyzed patient surveys and medical records for a population-based cohort with metastatic colorectal cancer treated in multiple regions and health systems in the United States (US). Eligible patients were diagnosed with metastatic colorectal cancer and initiated first-line chemotherapy after US Food & Drug Administration (FDA) bevacizumab approval in February 2004. First-line bevacizumab therapy was defined as receiving bevacizumab within 8 weeks of starting chemotherapy for metastatic colorectal cancer. We evaluated factors associated with first-line bevacizumab treatment using logistic regression.

**Results:**

Among 355 patients, 31% received first-line bevacizumab in the two years after FDA approval, including 26% of men, 41% of women, and 16% of those ≥ 75 years. Use rose sharply within 6 months after FDA approval, then plateaued. 20% of patients received bevacizumab in combination with irinotecan; 53% received it with oxaliplatin. Men were less likely than women to receive bevacizumab (adjusted OR 0.55; 95% CI 0.32-0.93; p = 0.026). Patients ≥ 75 years were less likely to receive bevacizumab than patients < 55 years (adjusted OR 0.13; 95% CI 0.04-0.46; p = 0.001).

**Conclusions:**

One-third of eligible metastatic colorectal cancer patients received first-line bevacizumab shortly after FDA approval. Most patients did not receive bevacizumab as part of the regimen used in the pivotal study leading to FDA approval.

## Background

Bevacizumab, a monoclonal antibody against vascular endothelial growth factor, was the first biologic agent shown to improve median overall survival (by 4.7 months) in patients with metastatic colorectal cancer, when given with cytotoxic chemotherapy [1]. Results of the first phase III study demonstrating this benefit were released publically in June, 2003, and published in June, 2004. The United States (US) Food and Drug Administration (FDA) approved bevacizumab for first-line treatment of metastatic colorectal cancer in February, 2004. The drug was approved by the European Union a year later in January, 2005. Although effective in prolonging survival, bevacizumab is expensive,[2] can cause hypertension, cardiovascular events, and in rare instances, severe hemorrhage or gastrointestinal tract perforation [1,3].

Little is known about the early uptake of new biologic therapies such as bevacizumab for advanced cancer. Specifically, 1) what is the initial dissemination pattern of biologic agents?; and 2) how do clinicians interpret clinical trial results for biologic agents, especially since selective trial eligibility might not represent the broad spectrum of patients treated in the community setting?[4] Registry studies for bevacizumab have examined post-marketing safety and efficacy, but these studies cannot answer our two questions since all enrolled patients received bevacizumab [5,6].

Answering these questions is critically important in light of recent regulatory decisions related to bevacizumab. At the end of 2010, the FDA recommended removing bevacizumab's breast cancer indication due to lack of survival benefit; the European Medicines Agency has maintained its indication but only in combination with paclitaxel. In light of the high drug cost, we must understand how novel agents are used in standard care since in certain instances, rapid dissemination occurs despite lack of long-term, phase IV data.

We used data from the Cancer Care Outcomes Research and Surveillance (CanCORS) Consortium, a US population- and healthcare systems-based study of newly-diagnosed patients with colorectal cancer, to describe the uptake of bevacizumab and to identify factors associated with its use. Comprehensive medical record abstraction enabled us to evaluate bevacizumab use in relationship to comorbidity and other patient characteristics not typically available in cancer registry data [7]. Thus, we were able to examine dissemination of this new therapy in a broad range of community settings.

## Methods

### Cancer Care Outcomes Research and Surveillance Consortium

CanCORS is a prospective, observational, population- and healthcare systems-based cohort study designed to determine how characteristics of cancer patients, providers, and health care organizations influence treatments and outcomes in newly diagnosed lung or colorectal cancer patients [8]. The full CanCORS cohort appears representative of the US Surveillance, Epidemiology, and End Results (SEER) registry sample: CanCORS participants did not differ from their corresponding SEER population by more than 8 percentage points in any important demographic characteristics such as gender, race, age, and stage [9]. Patients with colorectal cancer were nationally enrolled from geographically diverse populations and health care systems, including five integrated health care systems in the NCI-funded Cancer Research Network, and fifteen Veterans Administration Hospitals [8]. Primary data were collected from patient surveys and medical records. Human subject committees approved the study protocol at each participating site.

### Patients

**Figure 1:**
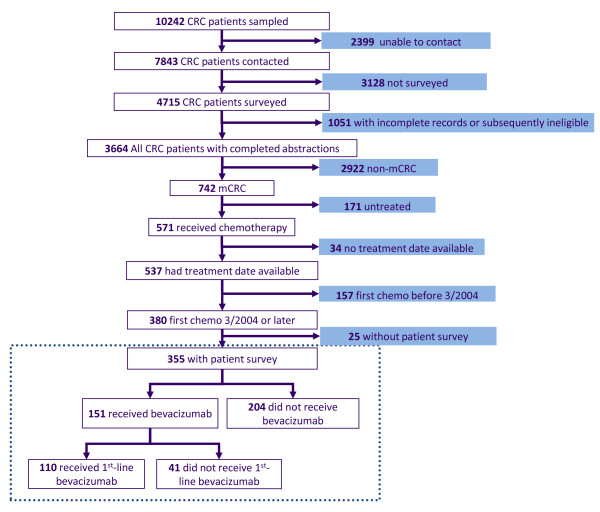
CONSORT-like diagram for the analytic cohort obtained from the overall CanCORS cohort*. *The dotted line encases patients who were considered for this analysis. First-line therapy with bevacizumab was defined as receipt of bevacizumab within 8 weeks of starting any chemotherapy for stage IV or recurrent colorectal cancer (mCRC).

Eligible patients were at least 21 years old and were identified within 3 months of diagnosis of colorectal cancer. Three thousand six hundred sixty-four incident cases of colorectal cancer (Figure 1) were enrolled in the CanCORS cohort between September, 2003 to January, 2006, including 742 patients (20%) with advanced colorectal cancer (677 with stage IV cancer at diagnosis, and 65 with a metastatic or regional recurrence within the 15-month post-diagnosis follow-up period of the study). Of note, this stage distribution (20% with advanced cancer) is consistent with that seen in the SEER registry, where patients with advanced colorectal cancer comprise 19% of all patients [10]. Of the 742 patients in our cohort with stage IV or recurrent disease, 355 initiated first-line treatment after the bevacizumab approval date in February, 2004 and were included in this analysis. Hence, the subcohort of interest for this analysis began treatment from March, 2004-January, 2006. All patients or their surrogates provided informed consent at enrollment. Recruitment materials and patient surveys were translated into Spanish and Chinese.

### Data Collection

Medical records data were abstracted by trained abstractors at each of the data-collection sites. The time window for records abstraction was three months before diagnosis to fifteen months after diagnosis. Data abstractors had professional experience as cancer registrars or nurses and underwent an intensive four-day training course on data collection processes and standards in CanCORS. Quality assurance was monitored by completion of up to six gold-standard reference chart abstractions per abstractor.

Presence of comorbid illness was collected as part of the medical record abstraction. The degree of comorbidity was scored using the Adult Comorbidity Evaluation 27 (ACE-27), a 27-item index developed to provide prognostic information for cancer patients [11].

Primary data were collected from patients via computer-assisted telephone interviews 4-7 months after diagnosis. For patients who had died or were too ill to be interviewed, a surrogate (relative or household member) familiar with their cancer care was interviewed. The surveys (available at http://www.cancors.org/public) assessed patients' sociodemographic characteristics (age, race/ethnicity, annual income), insurance coverage, comorbid conditions, and beliefs about cancer care (see Additional file 1) [12].

Surveys were the primary source of insurance information; medical records provided a secondary source. We categorized patients by whether or not they received care in an integrated health system, defined as one of the 5 participating health maintenance organizations, a California Kaiser Permanente plan, or one of the participating Veterans Administration hospitals [8]. Patients were also classified by US region (South, including sites in Alabama, Mississippi, Tennessee, Texas, Georgia; Atlantic, including sites in Massachusetts, Maryland, New York, and North Carolina; and West/Midwest, including sites in Arizona, California, Oregon, Washington, Iowa, Illinois, Indiana, Michigan, Minneapolis, Indiana).

First-line therapy with bevacizumab was defined as receipt of bevacizumab within 8 weeks of starting any chemotherapy for stage IV or recurrent colorectal cancer. For instance, a patient who began metastatic colorectal cancer treatment with cytotoxic chemotherapy, then had bevacizumab added to the regimen 6 weeks later was categorized as receiving first-line bevacizumab. Rates of first-line bevacizumab use pertain to the period from March, 2004 (the month after FDA approval) to January, 2006. To evaluate the relationship between diffusion of bevacizumab and calendar time since FDA approval, we analyzed quarterly usage. Since CanCORS participants were enrolled as a fixed cohort, and the rate of enrollment in CanCORS did not remain constant over the study period, the denominator of eligible patients per quarter (any patients starting any first-line chemotherapy) decreased over time.

The FDA approved bevacizumab based on the results of the pivotal phase III study where bevacizumab was delivered in combination with 5-fluorouracil and irinotecan;[1] however, approval was for use "with intravenous 5-fluorouracil-based chemotherapy," without specifying use of additional agents, such as irinotecan or oxaliplatin [13]. To determine how strictly practitioners interpreted trial data and FDA indication, we described which other drugs were delivered in combination with first-line bevacizumab.

### Statistical Analyses

We calculated descriptive statistics summarizing sociodemographic, comorbidity data, and survey-based patient preferences and beliefs. We evaluated factors associated with treatment using logistic regression. Results of multivariable modeling are presented as odds ratios (OR), two-sided p-values, and 95% confidence intervals (with associated confidence interval plots) [14]. Variables were selected for inclusion in multivariable models using a combination of statistical selection criteria (e.g., sufficient variation across values of the outcome, p < 0.25 in unadjusted analyses) and a priori scientific interest (race/ethnicity and comorbidity). Multiple imputation was used to address item nonresponse for survey-based variables and was performed centrally by the CanCORS Statistical Coordinating Center [15]. The presented results from multivariable models incorporate formal imputation adjustments [16]. Data analysis was conducted at the Durham Veterans Administration Medical Center, the coordinating site for Veterans Administration hospitals participating in CanCORS. This analysis used CanCORS core data (version 1.9), medical record data (version 1.9) and patient survey data (version 1.8). Statistical analyses were performed using SAS for Windows Version 9.2 (SAS Institute, Cary, NC).

## Results

### Cohort characteristics

Three hundred fifty-five patients met inclusion criteria for this analysis (Figure 1). Their characteristics are detailed in Table 1; 66% percent were male, 18% were ≥ 75 years old, and 62% were white. Seventy-three percent had no or mild comorbidity (ACE-27 score of 0-1). Since the most common contraindications to bevacizumab are related to cardiovascular disease, we described incidence of cardiovascular disease based on data from the ACE-27 (Table 1). Forty-nine percent of patients had at least one cardiovascular risk factor. Thirty-six percent had private insurance, 13% had public insurance, 27% had Medicare plus supplemental insurance, and 19% had Veterans Administration healthcare. Of those with only public insurance, 64% had Medicare, 62% had Medicaid, and 33% had both.

**Table 1:** Baseline characteristics of study cohort

Characteristic*	All patients (n = 355)	
	
	N	%
Age in years		

< 55	92	26

55-64	106	30

65-74	94	27

≥ 75	63	18

ACE-27 Comorbidity Index (score)		

None (0)	120	34

Mild (1)	140	39

Moderate (2)	51	14

Severe (3)	44	12

Race		

Non-white	134	38

White	221	62

Gender		

Female	120	34
Male	235	66

Insurance		

Missing	19	5

Public	45	13

Medicare + Supplemental	96	27

Veterans Administration	67	19

Private	128	36

Geographic region		

West/Midwest	178	50

South	91	26
Atlantic	86	24

Health system		

Fee-for-Service	212	60

Integrated health system	143	40

Annual household income		

Missing	66	19

< $20,000/year	100	28

≥ $20,000/year	189	53

Diagnosis of metastatic disease		

Recurrence	58	16

Stage IV at diagnosis	297	84

Primary tumor site		

Missing	5	1

Colon	258	73

Rectum	76	21
Colorectal	16	5

Receipt of surgery within 4 weeks of chemotherapy		

No qualifying surgery within 4 weeks	321	90

Qualifying surgery within 4 weeks	34	10

Presence of any of following 5 cardiovascular risks		

No risk factor grade > 0	181	51

Presence of ≥ 1 risk factor grade > 0	174	49

Hypertension		

Grade 0	203	57

Grade > 0	152	43

Angina/coronary artery disease		

Grade 0	307	87

Grade > 0	48	14

Venous disease including venous thrombosis		

Grade 0	346	98

Grade > 0	9	3

Peripheral artery disease		

Grade 0	345	97

Grade > 0	10	3

Congestive heart failure		

Grade 0	342	96

Grade > 0	13	4

*Only three patients had missing insurance data after imputation. The composition of health systems in the health system categorization of fee-for-services vs. integrated has been previously described [8]. Comorbidity was calculated using the Adult Comorbidity Evaluation-27 (ACE-27) index [11]. The cardiovascular comorbidities were collected as a part of the ACE-27. Some percentages may not sum to 100 due to rounding.

### Dissemination of bevacizumab after FDA approval

**Figure 2:**
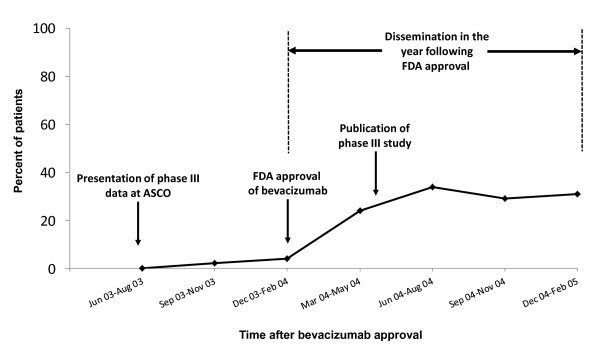
**Bevacizumab usage within 12 months of FDA approval for colorectal cancer**. ^. ^This study followed patients until January, 2006, but data in this figure is shown only until February 2005 as numbers of participants starting any first-line chemotherapy (denominator) within a particular quarter diminishes over time due to the fixed-cohort effect. In the year following approval, the highest percentage of use was seen in July, 2004 (12 of 25, 48%), a month after publication of the pivotal, phase III study which confirmed the drug's efficacy in the first line as a part of combination therapy.

Of the 355 patients in this cohort eligible to receive bevacizumab from March, 2004 (after FDA approval) to January, 2006, 31% received the drug as a component of their first-line systemic treatment for stage IV or recurrent disease (Figure 1). Figure 2 displays the quarterly use of bevacizumab 9 months before FDA approval to 12 months after FDA approval of bevacizumab. Only 20% of patients starting first line therapy for metastatic colorectal cancer received first-line bevacizumab in March 2004, the month after FDA approval. In the year following approval, the highest percentage of use was seen during June-August 2004, specifically in July 2004 (48%), a month after publication of the pivotal phase III study which reported the drug's efficacy [1].

### Drugs used in combination with bevacizumab

The FDA approved bevacizumab for use with intravenous 5-fluorouracil-based chemotherapy. Seventy-five percent of patients received bevacizumab with intravenous 5-fluorouracil (with or without other chemotherapy), in accordance with the FDA indication (Table 2). Thirteen percent received bevacizumab with capecitabine (with or without other chemotherapy).

**Table 2:** Drugs used in combination with bevacizumab*

	N (N = 110)	%
**Use as indicated by FDA label**		

5-fluorouracil included in the regimen	82	75

5-fluorouracil not included in the regimen	15	14

Unable to determine 5-fluorouracil use	13	12

**Chemotherapy give in combination with bevacizumab**		
5-fluorouracil plus oxaliplatin	51	46

5-fluorouracil plus irinotecan	21	19

Capecitabine plus oxaliplatin	8	7

Capecitabine plus irinotecan	1	1

5-fluorouracil alone	9	8

Capecitabine alone	5	5

Other	15	14

*Some percentages may not sum to 100 due to rounding. FDA, Food and Drug Administration.

Only 20% of those who received first-line bevacizumab received it with irinotecan--19% with 5-fluorouracil, as described in the pivotal phase III study, and 1% with capecitabine (Table 2). Fifty-three percent received bevacizumab in combination with oxaliplatin. Eight percent received bevacizumab with 5-fluorouracil alone; 5% with capecitabine alone; and 14% with "other" agents. No patients received first-line bevacizumab as a single agent.

### Factors associated with bevacizumab use

**Figure 3:**
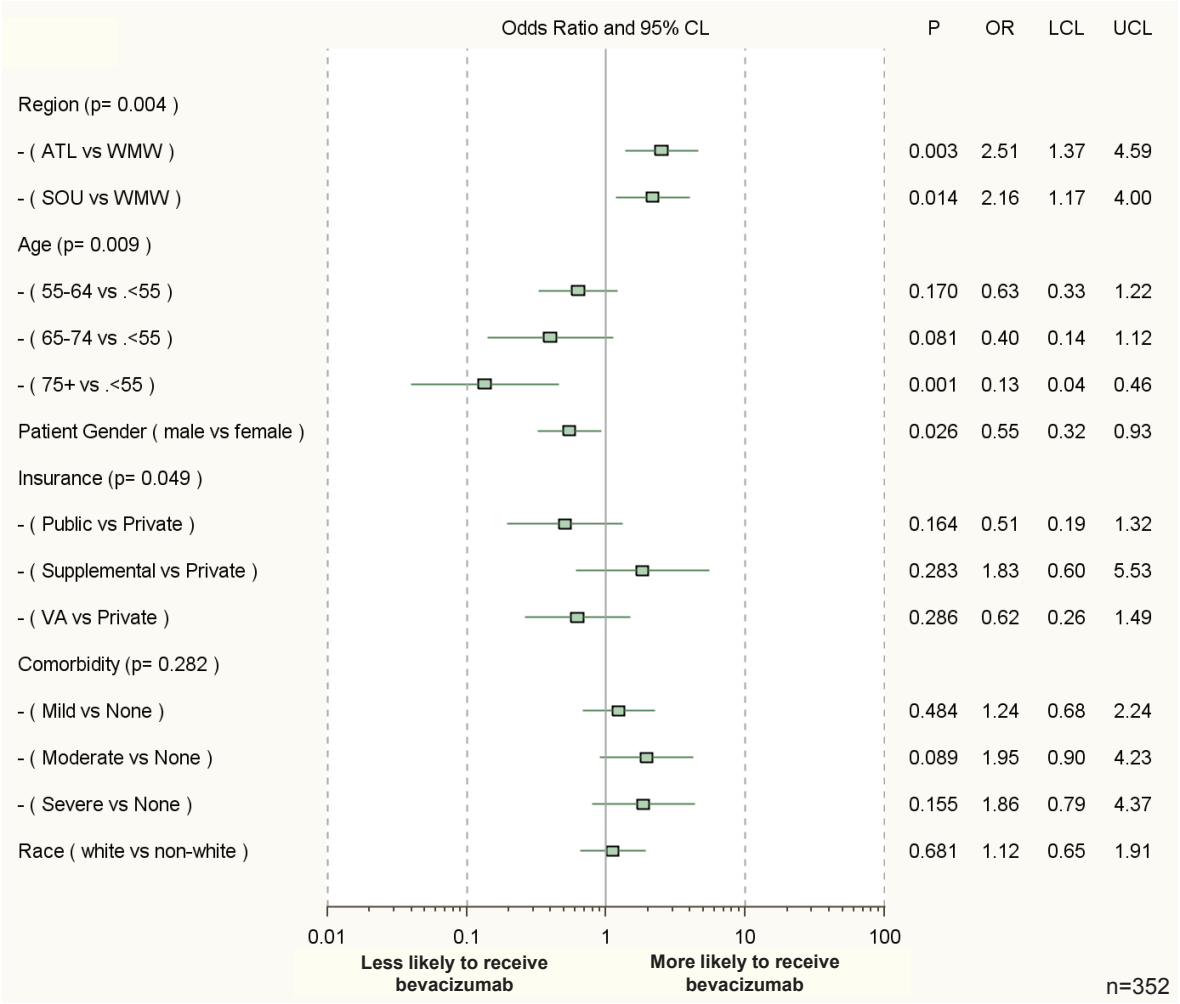
**Sociodemographic and clinical factors associated with receipt of first-line bevacizumab: multivariable logistic regression model results^#^**. ^#^n = 352. Three patients were excluded due to missing explanatory variables. Model results incorporate formal adjustments for multiple imputations of missing data. OR's and CI's are plotted on a log scale. Comorbidity was calculated using the Adult Comorbidity Evaluation-27 Scale. Abbreviations: OR, adjusted odds ratio; CI, confidence interval; ATL, Atlantic; SOU, Southern; WMW, West/Midwest. *P-values for aggregate variable effects (type 3 Wald statistics) are listed next to each variable category in the left-hand column.

Twenty-six percent of men received first-line bevacizumab (Table 3). In the multivariable model (Figure 3), men were significantly less likely to receive first-line bevacizumab than women (adjusted OR 0.55; 95% CI 0.32-0.93; p = 0.026). Bevacizumab use declined with age: first-line bevacizumab was used by 41% of those < 55 years of age, 32% of those 55-64 years of age, 30% of those 65-74 years of age, and 16% for those ≥ 75 years of age. Patients ≥ 75 years old were significantly less likely to receive first-line bevacizumab than younger (< 55 years) patients (adjusted OR 0.13; 95% CI 0.04-0.46; p = 0.001). Patients in the Atlantic region were more likely to receive bevacizumab than those in the West-Midwest (adjusted OR 2.51; 95% CI 1.37-4.59; p = 0.003), and patients in the South region also tended to receive bevacizumab more often than those in the West-Midwest (adjusted OR 2.16; 95% CI 1.17-4.00; p = 0.014).

**Table 3:** Percent of patients who received first-line bevacizumab by selected patient characteristics

Characteristic*	Received first-line bevacizumab (n = 110)	**Unadjusted p-value**^#^
	
	%	
Age (years)		0.007

< 55	41	

55-64	32	

65-74	30	

≥ 75	16	

ACE-27 Comorbidity Index (score)		0.831

None (0)	29	

Mild (1)	30	

Moderate (2)	35	

Severe (3)	34	

Race		0.403

Non-white	28	

White	33	

Gender		0.005

Female	41	

Male	26	

Insurance		0.005

Private	41	

Public	20	

Medicare + supplemental	32	

Veterans Administration	21	

Missing	21	

Health system		0.050

Fee-for-service	35	

Integrated health system	25	

Geographic region		0.003

West/Midwest	23	

Southern	35	

Atlantic	43	

Annual household income		0.127

< $20,000/year	25	

≥ $20,000/year	34	

Missing	30	

Diagnosis of metastatic disease		0.532

Stage IV at diagnosis	30	

Recurrence	35	

Did anyone mention that enrollment in a clinical trial might be an option for you?		0.152

Yes	38	

No	28	

Missing	35	

How often do you read books, magazines or newspapers?		0.111

Often	35	

Rarely	24	

Missing	26	

After talking with your doctors about chemotherapy, how likely did you think it was that chemotherapy would have side-effects or complications?		0.133

Likely	33	

Not likely	24	

Missing	30	

*The composition of health systems in the categorization of fee-for-services vs. integrated health systems has been previously described [8]. Comorbidity was calculated using the Adult Comorbidity Evaluation-27 (ACE-27) index [11]. The cardiovascular comorbidities were collected as a part of the ACE-27. ^#^Unadjusted p-values for aggregate variable effects (type 3 Wald statistics).

Patients' race was not significantly associated with receipt of bevacizumab, nor was insurance status. To assess the effect of organization of care, apart from insurance type, we substituted health system for insurance in the adjusted analyses. No significant association was seen between health system type and receipt of first-line bevacizumab, and none of the other associations were substantially altered. The relationship between patients' income and receipt of bevacizumab was not significant.

ACE-27 comorbidity score was not significantly associated with receipt of first-line bevacizumab. Since cardiovascular comorbidity presents a potential contraindication, we also examined the specific impact of cardiovascular comorbidity on bevacizumab use. In the unadjusted analysis, we found no statistically significant association between bevacizumab use and the presence of cardiovascular disease. Patients' knowledge, belief, and attitudes were also not statistically significantly associated with receipt of bevacizumab (Table 3). However, due to missingness, these variables were not included in the final analytic model.

## Discussion

Over the two years following approval of bevacizumab for first-line treatment of advanced colorectal cancer, we found that 31% of a nationally-representative sample of treated patients received the agent as a component of their care. Bevacizumab use showed an initial rapid uptake in the first 6 months after FDA approval and then leveled off. Seventy-five percent of patients received bevacizumab with intravenous 5-fluorouracil (with or without other chemotherapy), in accordance with the label. Only 19% of patients received bevacizumab with irinotecan and 5-fluorouracil, as described in the pivotal clinical trial which led to FDA approval. Prior to this study, little was known about the patterns of bevacizumab use soon after FDA approval, and little was known about the factors associated with early use. Registry studies for bevacizumab examined post-marketing safety and efficacy,[5,6] but all enrolled patients in those studies received bevacizumab; hence, those studies could not address questions related to dissemination.

The rapid increase noted in the first year was unprecedented for a cancer drug,[17] and was likely a consequence of two factors. First, when it was approved, the drug received a great deal of media attention, including being called "revolutionary."[18] This attention presumably promoted initial uptake by oncologists. Second, bevacizumab was the first anti-angiogenic agent to demonstrate improvement in overall survival for colorectal cancer-a potential paradigm shift in cancer treatment that also may have stimulated an upsurge of initial interest on the part of oncologists [19].

Why, despite the rapid rise, did early bevacizumab uptake level off at less than one-third of eligible patients? Our data suggest that clinicians may have restricted their use to a sub-segment of potentially eligible patients. We found that older age, even after adjusting for comorbidity, was associated with less bevacizumab use. The mean age of patients who received bevacizumab in our study was 58.2 years, nearly identical to the mean age of 59.5 years in the pivotal phase III trial [1]. Early adopters of the drug may have worried that its safety in elderly patients was not adequately addressed by the trial design and were hesitant to use the novel therapy in older patients without post-approval data. Hence, they limited use of the drug to patients who were similar to those treated in the definitive clinical trial. Similar findings have been noted among colorectal cancer patients eligible for adjuvant chemotherapy: the elderly receive lower doses and shorter duration of therapy than recommended by trial data [20].

Men were less likely than women to receive bevacizumab. It is unclear why gender played a role, especially since prior studies investigating the impact of gender on treatment of colorectal cancer have found no difference [21,22]. Men are more likely than women to have subclinical cardiovascular disease [23] and to be taking aspirin [24]. In the phase III trial of bevacizumab, patients were excluded if they had significant cardiovascular disease, regular aspirin use (> 325 mg per day), preexisting bleeding disorders, or full-dose anticoagulation [1]. Although we adjusted for cardiovascular comorbidity, our measures may not have fully captured these specific conditions. Clinicians might have been more hesitant to use the drug in men with greater cardiovascular risk given the exclusion criteria in the pivotal trial. To ensure that the gender effect was not due to confounding by the largely male Veterans Administration population we did a secondary analysis excluding the Veterans Administration patients and found no change in the association between gender and receipt of bevacizumab.

The FDA approved bevacizumab in 2004 to be used in combination with intravenous 5-fluorouracil-based chemotherapy,[13] based on results of the pivotal phase III study, which used bevacizumab in combination with irinotecan and 5-fluorouracil (IFL) [1]. Although the pivotal trial was designed with IFL as the chemotherapy backbone, the label considered bevacizumab in combination with any first-line 5-fluorouracil-containing regimen to be on label. The relative flexibility that the FDA provided to oncologists by labeling it for use in combination with any 5-fluorouracil-containing regimen might have been the result of rising concern over excessive rates of treatment-associated deaths with IFL [25,26]. Hence, oncologists could use bevacizumab in combination with oxaliplatin and still remain in accordance with the FDA indication.

Despite lack of evidence at the time for use with oxaliplatin, more than twice as many patients in our study received bevacizumab with oxaliplatin than with irinotecan. Due to the concern over treatment-associated deaths with IFL, clinicians may have opted to use oxaliplatin-based regimens with the assumption that the incremental advantage of adding bevacizumab would be similar to IFL. In fact, when evidence was subsequently published in 2008 for bevacizumab given in combination with 5-fluorouracil and oxaliplatin (FOLFOX), no significant improvement in overall survival was demonstrated over FOLFOX alone [27]. Additionally, some bevacizumab-receiving patients in our study were not treated with first-line 5-fluorouracil at all, thereby receiving off-label treatment. A few bevacizumab-receiving patients were treated with capecitabine, an approach that was not supported by published evidence or FDA indication.

We observed a somewhat complex relationship between the initial evidence supporting use of bevacizumab in patients with advanced colorectal cancer and early patterns of uptake. Clinicians were apparently guided by the eligibility criteria of the trial, hesitating to treat older patients or those at risk for cardiovascular compromise. However, they chose to give the drug in combination with chemotherapy regimens not supported by the FDA or existing evidence at the time. Future studies of the dissemination of subsequently approved targeted agents will be needed to determine whether these patterns were specific to adoption of this new biologic agent or apply more broadly to other biologic treatments for cancer. We also saw no association with patient preferences or beliefs. Our findings thus suggest that oncologists, not patients, were the primary decision-makers about whether to include bevacizumab for patients treated with chemotherapy.

Our analysis revealed regional disparities, with patients living in the Atlantic region being more likely than those in other regions to receive bevacizumab. Regional variation has been well-described in relation to delivery of both surgery and chemotherapy for various cancers [28-33]. Our findings of regional variation are unlikely the result of differential reimbursement for bevacizumab since the US Centers for Medicare & Medicaid Services (CMS) approved coverage for bevacizumab retroactive to the FDA approval date. Furthermore, the regional variation is unlikely to have been the result of formulary availability within each health system, since in the US anticancer drugs are generally available on formulary at the time of FDA approval. In their review of 59 anticancer drugs (including bevacizumab), Mason et al. found that 100% of drugs were covered by CMS and the Veterans Administration health system from the time of FDA approval [34]. Additionally, at least in the Veterans Administration health system, bevacizumab was available prior to formulary listing but after FDA approval via non-formulary request (personal communication, Geraci M, 2010).

Our study has several limitations. While we assessed patient preferences and beliefs, we could not assess preferences specific to bevacizumab. Our cohort is a small sub-sample drawn from a relatively large cohort, though it is large enough to support robust analysis, and CanCORS enrollees are representative of patients included in the SEER cancer registry [9]. Additionally, longitudinal follow-up of our cohort was short.

## Conclusions

We found a rapid uptake of bevacizumab after FDA approval, though only a third of eligible patients received the drug. Use of bevacizumab in practice differed from that described in clinical trials leading to FDA approval. Dissemination of novel agents might be improved through comparative effectiveness research measuring the benefits and safety of treatments through post-marketing (phase IV) studies that track the dissemination of new therapies and their associated outcomes.

## Competing interests

SYZ discloses receipt of honoraria from Genentech. PC discloses receipt of research funding from Genentech. Other authors have no conflicts of interest to disclose. All other authors declare no competing interests.

## Authors' contributions

SYZ, JLM, SCG, DHA, DS, JCW, MNF, JZA, and DP were responsible for conception and design. SYZ, SCG, DHA, JTL, LLZ, JCW, MNF, JZA, RW, KLK, PC, DWW, and DP assisted in collection and assembly of data. SYZ, JLM, SCG, DHA, DS, LLZ, JCW, MNF, JZA, RW, KLK, PAG, and DP participated in data analysis and interpretation. All authors participated in manuscript writing, and all authors approved the final manuscript.

## Funding

This work and the CanCORS Consortium are supported by grants from the National Cancer Institute to the Statistical Coordinating Center (grant number U01 CA093344) and the National Cancer Institute-supported Primary Data Collection and Research Centers (Dana-Farber Cancer Institute/Cancer Research Network (grant number U01 CA093332); Harvard Medical School/Northern California Cancer Center (grant number U01 CA093324); RAND/UCLA (grant number U01 CA093348); University of Alabama at Birmingham (grant number U01 CA093329); University of Iowa (grant number U01 CA093339); and the University of North Carolina (U01 CA 093326); the Agency for Healthcare Research Quality (grant number 03-438MO-03); and by a Department of Veterans Affairs grant to the Durham VA Medical Center (grant number HSRD CRS 02-164). The study sponsors (National Cancer Institute, the Agency for Healthcare Research Quality, and the Department of Veterans Affairs) played no role in study design, data collection, analysis, interpretation, writing, or decision to submit for publication.

## Pre-publication history

The pre-publication history for this paper can be accessed here:

http://www.biomedcentral.com/1471-2407/11/354/prepub

## Supplementary Material

Additional file 1 **Patient preference and belief variables that were not significantly related to receipt of chemotherapy**. Table. Click here for file

## References

[B1] Hurwitz H Fehrenbacher L Novotny W Cartwright T Hainsworth J Heim W Berlin J Baron A Griffing S Holmgren E Bevacizumab plus irinotecan, fluorouracil, and leucovorin for metastatic colorectal cancer N Engl J Med 2004 350 23 2335 2342 10.1056/NEJMoa032691 15175435

[B2] Schrag D The price tag on progress--chemotherapy for colorectal cancer N Engl J Med 2004 351 4 317 319 10.1056/NEJMp048143 15269308

[B3] Hurwitz H Saini S Bevacizumab in the treatment of metastatic colorectal cancer: safety profile and management of adverse events Semin Oncol 2006 33 5 Suppl 10 S26 34 17145522 10.1053/j.seminoncol.2006.08.001

[B4] Elting LS Cooksley C Bekele BN Frumovitz M Avritscher EB Sun C Bodurka DC Generalizability of cancer clinical trial results: prognostic differences between participants and nonparticipants Cancer 2006 106 11 2452 2458 10.1002/cncr.21907 16639738

[B5] Grothey A Sugrue MM Purdie DM Dong W Sargent D Hedrick E Kozloff M Bevacizumab Beyond First Progression Is Associated With Prolonged Overall Survival in Metastatic Colorectal Cancer: Results From a Large Observational Cohort Study (BRiTE) J Clin Oncol 2008 26 33 5326 5334 10.1200/JCO.2008.16.3212 18854571

[B6] Cohn AL Bekaii-Saab T Bendell JC Hurwitz H Kozloff M Roach N Tezcan H Feng S Sing A Grothey A Clinical outcomes in bevacizumab (BV)-treated patients (pts) with metastatic colorectal cancer (mCRC): Results from ARIES observational cohort study (OCS) and confirmation of BRiTE data on BV beyond progression (BBP) J Clin Oncol (Meeting Abstracts) 2010 28 15_suppl 3596

[B7] Ward E Halpern M Schrag N Cokkinides V DeSantis C Bandi P Siegel R Stewart A Jemal A Association of insurance with cancer care utilization and outcomes CA Cancer J Clin 2008 58 1 9 31 10.3322/CA.2007.0011 18096863

[B8] Ayanian JZ Chrischilles EA Wallace RB Fletcher RH Fouad MN Kiefe CI Harrington DP Weeks JC Kahn KL Malin JL1 Understanding Cancer Treatment and Outcomes: The Cancer Care Outcomes Research and Surveillance Consortium J Clin Oncol 2004 22 15 2992 2996 10.1200/JCO.2004.06.020 15284250

[B9] Representativeness of the CanCORS participants relative to Surveillance, Epidemiology & End Results (SEER) Cancer Registries, AcademyHealth Annual Research Meeting http://bcb.dfci.harvard.edu/catalano/representativenessAHabstrac15jan08.pdf

[B10] Fast Stats: An interactive tool for access to SEER cancer statistics http://seer.cancer.gov/statfacts/html/colorect.html

[B11] Piccirillo JF Tierney RM Costas I Grove L Spitznagel EL Jr Prognostic importance of comorbidity in a hospital-based cancer registry JAMA 2004 291 20 2441 2447 10.1001/jama.291.20.2441 15161894

[B12] Malin J Ko C Ayanian J Harrington D Nerenz D Kahn K Ganther-Urmie J Catalano P Zaslavsky A Wallace R Understanding cancer patients' experience and outcomes: development and pilot study of the Cancer Care Outcomes Research and Surveillance patient survey Supp Care Cancer 2006 14 8 837 848 10.1007/s00520-005-0902-8 16482448

[B13] Bevacizumab prescribing information http://www.accessdata.fda.gov/drugsatfda_docs/label/2009/125085s0168lbl.pdf

[B14] Galbraith RF A note on graphical presentation of estimated odds ratios from several clinical trials Stat Med 1988 7 8 889 894 10.1002/sim.4780070807 3413368

[B15] He Y Zaslavsky AM Harrington DP Catalano PJ Landrum MB Imputation in a multiformat and multiwave survey of cancer care Proc Joint Stat Meetings Am Stat Assoc 2007 1541 1549

[B16] Little RJ Rubin DB Statistical Analysis with Missing Data 2002 2 Hoboken, NJ: Wiley-Interscience

[B17] Muhsin M Graham J Kirkpatrick P Bevacizumab Nat Rev Drug Discov 2004 3 12 995 996 10.1038/nrd1601 15645606

[B18] Good News About Cancer. Seriously http://www.businessweek.com/technology/content/jun2004/tc2004063_2435_tc024.htm

[B19] Glassman RH Sun AY Biotechnology: identifying advances from the hype Nat Rev Drug Discov 2004 3 2 177 183 10.1038/nrd1309 15040581

[B20] Kahn KL Adams JL Weeks JC Chrischilles EA Schrag D Ayanian JZ Kiefe CI Ganz PA Bhoopalam N Potosky AL Adjuvant Chemotherapy Use and Adverse Events Among Older Patients With Stage III Colon Cancer JAMA 2010 303 11 1037 1045 10.1001/jama.2010.272 20233821 PMC2893553

[B21] Hodgson DC Fuchs CS Ayanian JZ Impact of Patient and Provider Characteristics on the Treatment and Outcomes of Colorectal Cancer J Natl Cancer Inst 2001 93 7 501 515 10.1093/jnci/93.7.501 11287444

[B22] Ayanian JZ Zaslavsky AM Fuchs CS Guadagnoli E Creech CM Cress RD O'Connor LC West DW Allen ME Wolf RE Use of Adjuvant Chemotherapy and Radiation Therapy for Colorectal Cancer in a Population-Based Cohort J Clin Oncol 2003 21 7 1293 1300 10.1200/JCO.2003.06.178 12663717

[B23] Robinson J Fox K Bullano M Grandy S Group tSS Atherosclerosis profile and incidence of cardiovascular events: a population-based survey BMC Cardiovasc Dis 2009 9 1 46 10.1186/1471-2261-9-46 PMC2753582 19754940

[B24] Cannon CP Rhee KE Califf RM Boden WE Hirsch AT Alberts MJ Cable G Shao M Ohman EM Steg PG Current Use of Aspirin and Antithrombotic Agents in the United States Among Outpatients With Atherothrombotic Disease (from the REduction of Atherothrombosis for Continued Health [REACH] Registry) Am J Cardiol 2010 105 4 445 452 10.1016/j.amjcard.2009.10.014 20152237

[B25] Sargent DJ Niedzwiecki D O'Connell MJ Schilsky RL Recommendation for Caution with Irinotecan, Fluorouracil, and Leucovorin for Colorectal Cancer N Engl J Med 2001 345 2 144 146 11450666 10.1056/NEJM200107123450213

[B26] Rothenberg ML Meropol NJ Poplin EA Van Cutsem E Wadler S Mortality Associated With Irinotecan Plus Bolus Fluorouracil/Leucovorin: Summary Findings of an Independent Panel J Clin Oncol 2001 19 18 3801 3807 11559717 10.1200/JCO.2001.19.18.3801

[B27] Saltz LB Clarke S Diaz-Rubio E Scheithauer W Figer A Wong R Koski S Lichinitser M Yang TS Rivera F Bevacizumab in combination with oxaliplatin-based chemotherapy as first-line therapy in metastatic colorectal cancer: a randomized phase III study J Clin Oncol 2008 26 12 2013 2019 10.1200/JCO.2007.14.9930 18421054

[B28] Govindarajan A Coburn NG Kiss A Rabeneck L Smith AJ Law CHL Population-Based Assessment of the Surgical Management of Locally Advanced Colorectal Cancer J Natl Cancer Inst 2006 98 20 1474 1481 10.1093/jnci/djj396 17047196

[B29] Krupski TL Kwan L Afifi AA Litwin MS Geographic and Socioeconomic Variation in the Treatment of Prostate Cancer J Clin Oncol 2005 23 31 7881 7888 10.1200/JCO.2005.08.755 16204005

[B30] Nattinger AB Gottlieb MS Veum J Yahnke D Goodwin JS Geographic variation in the use of breast-conserving treatment for breast cancer N Engl J Med 1992 326 17 1102 1107 10.1056/NEJM199204233261702 1552911

[B31] Polsky D Armstrong KA Randall TC Ross RN Even-Shoshan O Rosenbaum PR Silber JH Variation in Chemotherapy Utilization in Ovarian Cancer: The Relative Contribution of Geography Health Services Res 2006 41 6 2201 2218 10.1111/j.1475-6773.2006.00596.x PMC1955308 17116116

[B32] Smith GL Xu Y Shih Y-CT Giordano SH Smith BD Hunt KK Strom EA Perkins GH Hortobagyi GN Buchholz TA Breast-Conserving Surgery in Older Patients with Invasive Breast Cancer: Current Patterns of Treatment Across the United States J Am Coll Surg 2009 209 4 425 433 e422 10.1016/j.jamcollsurg.2009.06.363 19801315

[B33] Fairfield KM Lucas FL Earle CC Small L Trimble EL Warren JL Regional variation in cancer-directed surgery and mortality among women with epithelial ovarian cancer in the medicare population Cancer 2010 116 20 4840 4848 10.1002/cncr.25242 20578182

[B34] Mason A Drummond M Ramsey S Campbell J Raisch D Comparison of Anticancer Drug Coverage Decisions in the United States and United Kingdom: Does the Evidence Support the Rhetoric? J Clin Oncol 2010 28 20 3234 3238 10.1200/JCO.2009.26.2758 20498408

