# Supplementary material for: Early dissemination of bevacizumab for advanced colorectal cancer: a prospective cohort study
Source: BMC Cancer. 2011 Aug 16;11:354. doi: 10.1186/1471-2407-11-354 (PMC3174931; doi:10.1186/1471-2407-11-354)
Supplement: Additional file 1 — Patient preference and belief variables that were not significantly related to receipt of chemotherapy. Table. [file 1471-2407-11-354-S1.DOC]

**Additional file 1.** Patient preference and belief variables that were not significantly related to receipt of chemotherapy.

| Marital status | Do you worry about the cost of treatment? |
| --- | --- |
| Education | Will treatment help you live longer? |
| EQ5D | Will treatment cure your cancer? |
| Will chemotherapy cure your cancer? | Will treatment help you feel better? |
| Preference for extending life or relieving pain? | Does your doctor listen to you? |
| Quality of your care compared to others with cancer | Does your doctor explain? |
| Preference for treatment that costs less or extends life? | Does your doctor give you enough information? |
| What is your actual decision-making role? | Does your doctor encourage asking questions? |
| What is your preference for decision-making role? | Does your doctor respect you? |
| How long do you think you will live? | What is the quality of your chemotherapy care? |
| Did cost prevent testing/treatment for your cancer? | What is your family’s role in decision-making? |
| Do you read often? | Did your doctor mention a clinical trial? |
| Does cancer treatment cause side effects? | Do you worry about transportation to treatment? |
| Does treatment take time away from family? |  |
